# Face Detection Ensemble with Methods Using Depth Information to Filter False Positives

**DOI:** 10.3390/s19235242

**Published:** 2019-11-28

**Authors:** Loris Nanni, Sheryl Brahnam, Alessandra Lumini

**Affiliations:** 1 Department of Information Engineering, University of Padova, Via Gradenigo, 6, 35131 Padova, Italy; nanni@dei.unipd.it; 2 Department of Information Technology and Cybersecurity, Missouri State University, 901 S. National Street, Springfield, MO 65804, USA; 3 Dipartimento di Informatica—Scienza e Ingegneria, Università di Bologna, Via Sacchi 3, 47521 Cesena, Italy; alessandra.lumini@unibo.it

**Keywords:** face detection, depth map ensemble, filtering

## Abstract

A fundamental problem in computer vision is face detection. In this paper, an experimentally derived ensemble made by a set of six face detectors is presented that maximizes the number of true positives while simultaneously reducing the number of false positives produced by the ensemble. False positives are removed using different filtering steps based primarily on the characteristics of the depth map related to the subwindows of the whole image that contain candidate faces. A new filtering approach based on processing the image with different wavelets is also proposed here. The experimental results show that the applied filtering steps used in our best ensemble reduce the number of false positives without decreasing the detection rate. This finding is validated on a combined dataset composed of four others for a total of 549 images, including 614 upright frontal faces acquired in unconstrained environments. The dataset provides both 2D and depth data. For further validation, the proposed ensemble is tested on the well-known BioID benchmark dataset, where it obtains a 100% detection rate with an acceptable number of false positives.

## 1. Introduction

One of the most fundamental yet difficult problems in computer vision and human–computer interaction is face detection, the object of which is to detect and locate all faces within a given image or video clip. Face detection is fundamental in that it serves as the basis for many applications [1] that involve the human face, such as face alignment [2,3], face recognition/authentication [4,5,6,7], face tracking and tagging [8], etc. Face detection is a hard problem because unlike face localization, no assumptions can be made regarding whether any faces are located within an image [9,10]. Moreover, faces vary widely based on gender, age, facial expressions, and race, and can dramatically change in appearance depending on such environmental conditions as illumination, pose (out-of-plane rotation), orientation (in-plane rotation), scale, and degree of occlusion and background complexity. Not only must a capable and robust face detection system overcome these difficulties, but for many of today’s applications, it must also be able to do so in real time.

These challenges have resulted in a large body of literature reporting different methods for tackling the problem of face detection [11]. Yang et al. [12], who published a survey of face detection algorithms developed in the last century, have divided these earlier algorithms into four categories: knowledge-based methods, feature invariant approaches, template-matching methods, and appearance-based methods, the latter demonstrating some superiority compared with the other algorithms thanks to the rise in computing power. In general, these methods formulate face detection as a two-class pattern recognition problem that divides a 2D image into subwindows that are then classified as either containing a face or not [13]. Moreover, these approaches take a monocular perspective in the sense that they forgo any additional sensor or contextual information that might be available.

Around the turn of the century, Viola and Jones [14] presented a 2D detection method that has since become a major source of inspiration for many subsequent face detectors. The famous Viola–Jones (VJ) algorithm achieved real-time object detection using three key techniques: an integral image stratagem for efficient Haar feature extraction, a boosting algorithm (AdaBoost) for an ensemble of weak classifiers, and an attentional cascade structure for fast negative rejection. However, there are some significant limitations to the VJ algorithm that are due to the suboptimal cascades, the considerable pool size of the Haar-like features, which makes training extremely slow, and the restricted representational capacity of Haar features to handle, for instance, variations in pose, illumination, facial expression, occlusions, makeup, and age-related factors [15]. These problems are widespread in unconstrained environments, such as those represented in the Face Detection Dataset and Benchmark (FDDB) [16] where the VJ method fails to detect most faces [17].

Some early Haar-like extensions and enhancements intended to overcome some of these shortcomings include rotated Haar-like features [18], sparse features [19], and polygon features [20]. Haar-like features have also been replaced by more powerful image descriptors, such as local binary patterns (LBP) [21], spatial histogram features [22], histograms of oriented gradients (HoG) [23], multidimensional local Speeded-Up Robust Features (SURF) patches [24], and, more recently, by normalized pixel difference (NPD) [17] and aggregate channel features [25], to name but a few. 

Some older feature selection and filtering techniques for reducing the pool size, speeding up training, and improving the underlying boosting algorithm of the cascade paradigm include the works of Brubaker et al. [26] and Pham et al. [27]. In Küblbeck et al. [28], the illumination invariance and speed were improved with boosting combined with modified census transform (MCT); in Huang et al. [29], a method for detecting faces with arbitrary rotation in-plane and rotation off-plane angles in both still images and videos is proposed. For an excellent survey of face detection methods prior to 2010, see [11].

Some noteworthy 2D approaches produced in the last decade include the work of Li et al. [15] at Intel labs, who introduced a two-pronged strategy for the faster convergence speed of the SURF cascade, first by adopting, as with [24], multidimensional SURF features rather than single-dimensional Haar features to describe local patches, and second, by replacing decision trees with logistic regression. Two simple approaches that are also of note are those proposed in Mathias et al. [30], which obtained top performance compared with such commercial face detectors as Google Picasa, Face.com, Intel Olaworks, and Face++. One method is based on rigid templates, which is similar in structure to the VJ algorithm, and the other detector uses a simple deformable part model (DPM), which, in brief, is a generalizable object detection approach that combines the estimation of latent variables for alignment and clustering at the training time with multiple components and deformable parts to manage intra-class variance.

Four 2D models of interest in this study are the face detectors proposed by Nilsson et al. [31], Asthana et al. [32], Liao et al. [33], and Markuš et al. [34]. Nilsson et al. [31] used successive mean quantization transform (SMQT) features that they applied to a Split up sparse Network of Winnows (SN) classifier. Asthana et al. [32] employed face fitting, i.e., a method that models a face shape with a set of parameters for controlling a facial deformable model. Markuš et al. [34] combined a modified VJ method with an algorithm for localizing salient facial landmark points. Liao et al. [33], in addition to proposing the aforementioned scale-invariant NPD features, expanded the original VJ tree classifier with two leaves to a deeper quadratic tree structure.

Another powerful approach for handling the complexities of 2D face detection is deep learning [35,36,37,38,39,40,41]. For instance, Girshick et al. [36] were one of the first to use Convolutional Neural Networks (CNN) in combination with regions for object detection. Their model, appropriately named Region-CNN (R-CNN), consists of three modules. In the testing phase, R-CNN generates approximately 2000 category-independent region proposals (module 1), extracts a fixed-length deep feature vector from each proposal using a CNN (module 2), and then classifies them with Support Vector Machines (SVMs) (module 3). In contrast, the deep dense face detector (DDFD) proposed by Farfade et al. [37] requires no pose/landmark annotations and can detect faces in many orientations using a single deep learning model. Zhang et al. [39] proposed a deep learning method that is capable of extracting tiny faces, also using a single deep neural network.

Motivated by the development of affordable depth cameras, another way to enhance the accuracy of face detection is to go beyond the limitations imposed by the monocular 2D approach and include additional 3D information, such as that afforded by the Minolta Vivid 910 range scanner [42], the MU-2 stereo imaging system [43], the VicoVR sensor, the Orbbec Astra, and Microsoft’s Kinect [44], the latter of which is arguably the most popular 3D consumer-grade device on the market. Kinect combines a 2D RGB image with a depth map (RGB-D) that initially (Kinect 1) was computed based on the structured light principle of projecting a pattern onto a scene to determine the depth of every object but which later (Kinect 2) exploited the time-of-flight principle to determine depth by measuring the changes that an emitted light signal encounters when it bounces back from objects.

Since depth information is insensitive to pose and changes in illumination [45], many researchers have explored depth maps and other kinds of 3D information [46]; furthermore, several benchmark datasets using Kinect have been developed for both face recognition [44] and face detection [47]. The classic VJ algorithm was adapted to consider depth and color information a few years after Viola and Jones published their groundbreaking work [48,49]. To improve detection rates, most 3D face detection methods combine depth images with 2D gray-scale images. For instance, in Shieh et al. [50], the VJ algorithm is applied to images to detect a face, and then its position is refined via structured light analysis.

Expanding on the work of Shotton et al. [51], who used pair-wise pixel comparisons in depth images to quickly and accurately classify body joints and parts from single depth images for pose recognition, Mattheij et al. [52] compared square regions in a pair-wise fashion for face detection. Taking cues from biology, Jiang et al. [53] integrated texture and stereo disparity information to filter out locations unlikely to contain a face. Anisetti et al. [54] located faces by applying a course detection method followed by a technique based on a 3D morphable face model that improves accuracy by reducing the number of false positives, and Taigman et al. [6] found that combining a 3D model-based alignment with DeepFace trained on the Labeled Faces in the Wild (LFW) dataset [55] generalized well in the detection of faces in an unconstrained environment. Nanni et al. [9] overcame the problem of increased false positives when combining different face detectors in an ensemble by applying different filtering steps based on information in the Kinetic depth map.

The face detection system proposed in this paper is composed of an ensemble of face detectors that utilizes information extracted from the 2D image and depth maps obtained by Microsoft’s Kinect 1 and Kinect 2 3D devices. The goal of this paper, which improves the method presented in [9], is to test a set of filters, which includes a new wave-based filter proposed here, on a new collection of face detectors. The main objective of this study is to find those filters that preserve the ensemble’s increased rate of true positives while simultaneously reducing the number of false positives. Creating an ensemble of classifiers is a feasible method for improving performance in face detection (see [9]), as well as in many other classification problems. The main reason that ensembles improve face detection performance is that the combination of different methods increases the number of candidate windows and thus the probability of including a previously lost true positive. However, the main drawback of using ensembles in face detection is the increased generation of false positives. The rationale behind the proposed approach is to use some filtering steps to reduce false positives. The present work extends [9] by adding to the proposed ensemble additional face detectors.

The best performing system developed experimentally in this work is validated on the challenging dataset presented in [9] that contains 549 samples with 614 upright frontal faces. This dataset includes depth images as well as 2D images. The results in the experimental section demonstrate that the filtering steps succeed in significantly decreasing the number of false positives without significantly affecting the detection rate of the best-performing ensemble of face detectors. To validate the strength of the proposed new even system further, we validate it on the widely used BioID dataset [56], where it obtains a 100% detection rate with a limited number of false positives. Our best ensemble/filter combination outperforms the method proposed by Markuš et al. [34], which has been shown to surpass the performance of these well-known state-of-the-art commercial face detection systems: Google Picasa, Face++, and Intel Olaworks.

The organization of this paper is as follows. In Section 2, the strategy taken in this work for face detection is described along with the face detectors tested in the ensembles and the different filtering steps. In Section 3, the experiments on the two above-mentioned datasets are presented, along with a description of the datasets, definition of the testing protocols, and a discussion of the experimental results. The paper concludes, in Section 4, by providing a summary with some notes regarding future directions. The MATLAB code developed for this paper, along with the dataset, is freely available at https://github.com/LorisNanni.

## 2. Materials and Methods

**Figure 1:**
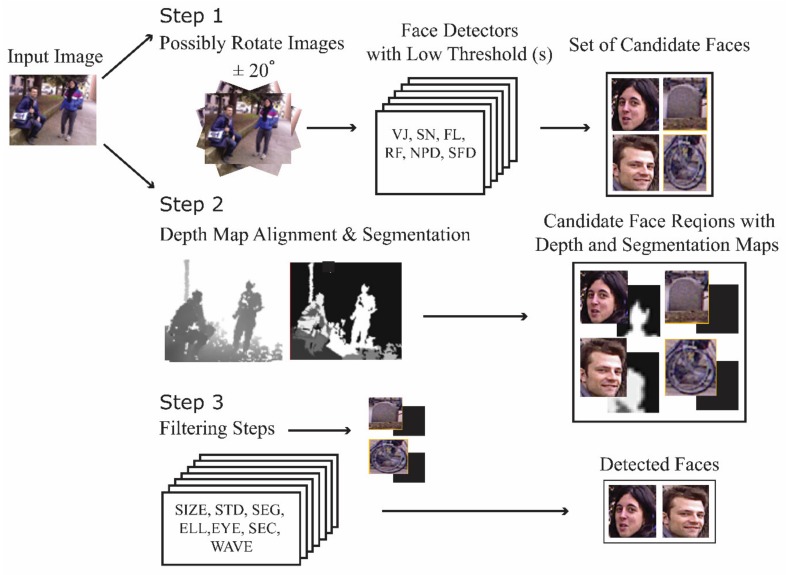
Schematic of the proposed face detection system.

The basic strategy taken in this work is to develop experimentally a high-performing face detection ensemble composed of well-known face detectors. The goal is to obtain superior results without significantly increasing the number of false positives. The system proposed here, as illustrated in Figure 1, is a three-step process.

In Step 1, high recall is facilitated by first performing face detection on the color images. A set of six face detectors (experimentally derived, as described in the experimental section) are applied to each image. The face detection algorithms tested in this paper are described in Section 2.2. Before detection, as also illustrated in Figure 1, color images are sometimes rotated {20°, −20°} to handle faces that are not upright. The addition of rotated images is noted in the experimental section whenever these are included in the dataset.

Since this first step is imprecise and therefore produces many false positives, the purpose of Step 2 is to align the depth maps to the color images so that false positives can be winnowed out in Step 3 by applying seven filtering approaches that take advantage of the depth maps. Alignment is accomplished by first calibrating the color and depth data using the calibration technique proposed in Herrera et al. [57]. The positions of the depth samples in 3D space are determined using the intrinsic parameters (focal length and principal point) of the depth camera. Then, these positions are reprojected in 2D space by considering both the color camera’s intrinsic parameters and the extrinsic parameters of the camera pair system. Next, color and depth values are associated with each sample, as described in Section 2.1. This operation is applied only to regions containing a candidate face to reduce computation time. Finally, in Step 3, these regions are filtered, as detailed in Section 2.3, to remove false positives from the candidate faces.

### 2.1. Depth Map Alignment and Segmentation

The color images and depth maps are jointly segmented by a procedure similar to that described in Mutto et al. [58] that has two main stages. In Stage 1, each sample is transformed into a six-dimensional vector. In Stage 2, the point set is clustered using the mean shift algorithm [59].

Every sample in the Kinetic depth map corresponds to a 3D point, $p_{i},\;i=1,\ldots ,N$, with N the number of points. The joint calibration of the depth and color cameras, as described in [57], allows a reprojection of the depth samples over the corresponding pixels in the color image so that each point is associated with the 3D spatial coordinates (x, y, and z) of $p_{i}$ and its RGB color components. Since these two representations lie in entirely different spaces, they cannot be compared directly, and all components must be comparable to extract multidimensional vectors that are appropriate for the mean shift clustering algorithm. Thus, a conversion is performed so that the color values lie in the CIELAB uniform color space, which represents color in three dimensions expressed by values representing lightness (L) from black (0) to white (100), a value (a) from green (−) to red (+), and a value (b) from blue (−) to yellow (+). This introduces a perceptual significance to the Euclidean distance between the color vectors that can be used in the mean shift algorithm.

Formally, the color information of each scene point in the CIELAB color space, c, can be described with the 3D vector:

(1) $$p_{i}^{c}=\left[\begin{array}{c} L\left(p_{i}\right)\\ a\left(p_{i}\right)\\ b\left(p_{i}\right) \end{array}\right],\;\;\;\;\;\;i=1,\;\ldots ,\;N.$$

The geometry, g, can be represented simply by the 3D coordinates of each point, thus:

(2) $$p_{i}^{g}=\left[\begin{array}{c} x\left(p_{i}\right)\\ y\left(p_{i}\right)\\ z\left(p_{i}\right) \end{array}\right],\;\;\;\;\;\;i=1,\;\ldots ,\;N.$$

The scene segmentation algorithm needs to be insensitive to the relative scaling of the point-cloud geometry. Moreover, the geometry and color distances must be brought into a consistent framework. For this reason, all the components of $p_{i}^{g}$ are normalized with respect to the average of the standard deviations of the point coordinates in the three dimensions $\sigma_{g}=\left(\sigma_{x}+\sigma_{y}+\sigma_{z}\right)/3$. Normalization produces the vector:

(3) $$\left[\begin{array}{c} \bar{x}\left(p_{i}\right)\\ \bar{y}\left(p_{i}\right)\\ \bar{z}\left(p_{i}\right) \end{array}\right]=\frac{3}{\sigma_{x}+\sigma_{y}+\sigma_{z}}\left[\begin{array}{c} x\left(p_{i}\right)\\ y\left(p_{i}\right)\\ z\left(p_{i}\right) \end{array}\right]=\frac{1}{\sigma_{g}}\left[\begin{array}{c} x\left(p_{i}\right)\\ y\left(p_{i}\right)\\ z\left(p_{i}\right) \end{array}\right].$$

To balance the relevance of color and geometry in the merging process, the color information vectors are normalized as well. The average of the standard deviations of the L, a, and b color components are computed producing the final color representation:

(4) $$\left[\begin{array}{c} \bar{L}\left(p_{i}\right)\\ \bar{a}\left(p_{i}\right)\\ \bar{b}\left(p_{i}\right) \end{array}\right]=\frac{3}{\sigma_{L}+\sigma_{a}+\sigma_{b}}\left[\begin{array}{c} L\left(p_{i}\right)\\ a\left(p_{i}\right)\\ b\left(p_{i}\right) \end{array}\right]=\frac{1}{\sigma_{c}}\left[\begin{array}{c} L\left(p_{i}\right)\\ a\left(p_{i}\right)\\ b\left(p_{i}\right) \end{array}\right].$$

Once the geometry and color information vectors are normalized, they can be combined for a final representation f:

(5) $$p_{i}^{f}=\left[\begin{array}{c} \begin{array}{c} \bar{L}\left(p_{i}\right)\\ \bar{a}\left(p_{i}\right)\\ \bar{b}\left(p_{i}\right) \end{array}\\ \begin{array}{c} \lambda_{\bar{x}}\\ \lambda_{\bar{y}}\\ \lambda_{\bar{z}} \end{array} \end{array}\right],$$

with the parameter λ adjusting the contribution to the final segmentation of color (low values of λ indicating high color relevance) and geometry (low values indicating high geometry relevance). By adjusting λ, the algorithm can be reduced to a color-based segmentation (λ=0) or to a geometry (depth)-only segmentation (λ→∞) (see [58] for a discussion of the effects that this parameter produces and for automatically tuning λ to an optimal value).

**Figure 2:**
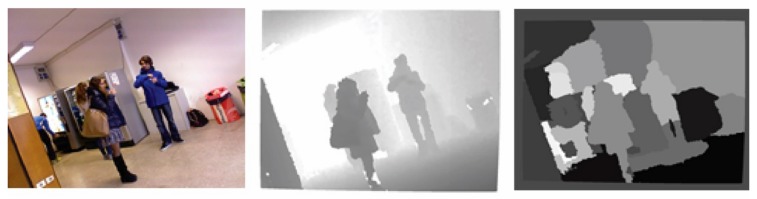
Color image (**left**), depth map (**middle**), and segmentation map (**right**).

Once the final vectors $p_{i}^{f}$ are calculated, they can be clustered by the mean shift algorithm [59] to segment the acquired scene. This algorithm offers an excellent trade-off between segmentation accuracy and computational complexity. For final refinement, regions are removed that are smaller than a predefined threshold, since they are typically due to noise. In Figure 2, examples of a segmented image are shown.

### 2.2. Face Detectors

We perform experiments on the fusion of six face detectors: the four detectors tested in [9] (the canonic VJ algorithm [14], a method using the Split up sparse Network of Winnows (SN) classifier [31], a modification of the VJ algorithm with fast localization (FL) [34], and a face detector based on Discriminative Response Map Fitting (DRMF) [32]), as well as two additional face detectors (the VJ modification using NPD features (NPD) [33] and a high-performance method implemented here: http://dlib.net/face_detector.py.html. In the following, this latter method is called Single Scale-invariant Face Detector (SFD). Each of these face detection algorithms is briefly described below.

#### 2.2.1. VJ

The canonical VJ algorithm [14] is based on Haar wavelets extracted from the integral image. Classification is performed, as noted in the introduction, by combining an ensemble of AdaBoost classifiers that select a small number of relevant descriptors with a cascade combination of weak learners. 

The disadvantage of this approach is that it requires considerable training time. However, it is relatively fast during the testing phase. The precision of VJ relies on the threshold s, which is used to classify a face within an input subwindow.

#### 2.2.2. SN

SN [31], available in MATLAB (http://www.mathworks.com/matlabcentral/fileexchange/loadFile.do?objectId=13701&objectType=FILE), feeds SMQT features, as briefly discussed in the Introduction, to a Split up Sparse Network of Winnows (SN) classifier. SMQT enhances gray-level images. This enhancement reveals the structure of the data and additionally removes some negative properties such as gain and bias. This is how SMQT features overcome to some extent the illumination and noise problem. 

SMQT features are extracted by moving a patch across the image while repeatedly downscaling and resizing it to detect faces of different sizes. The detection task is performed by the SN classifier, i.e., a sparse network of linear units over a feature space that can be used to create lookup tables.

#### 2.2.3. FL

FL (Fast Localization) [34] is a method that combines a modification of the standard VJ algorithm with a component for localizing a salient facial landmark. An image is scanned with a cascade of binary classifiers that considers a set of reasonable positions and scales. Computing a data structure, such as integral images, an image pyramid, or HoG features, etc., is not required with this method. An image region is classified as having a face when all the classifiers are in agreement that the region contains one. At this stage, another ensemble calculates the position of each facial landmark point. Each binary classifier in the cascade is an ensemble of decision trees that have pixel intensity comparisons in their internal nodes as binary tests. Moreover, they are based on the same feature type, unlike the VJ algorithm that uses five types of Haar-like features. Learning takes place with a greedy regression tree construction procedure and a boosting algorithm.

#### 2.2.4. RF

RF [32] is a face detector based on Discriminative Response Map Fitting (DRMF), which is a specific face fitting technique. DRMF is a discriminative regression method for the Constrained Local Models (CLMs) framework. Precision is adjusted in RF using the sensitivity parameter s that sets both a lower and a higher sensitivity value.

#### 2.2.5. NPD

NPD [33] extracts the illumination and blur invariant NPD features mentioned in the Introduction. NPD is computed as the difference-to-sum ratio between two pixels and is extremely fast because it requires only one memory access using a lookup table. However, because NPD contains redundant information, AdaBoost is applied to select the most discriminative feature set and to construct strong classifiers. The Gentle AdaBoost algorithm [60] is adopted for the deep quadratic trees. The splitting strategy consists in quantizing the feature range into l discrete bins (l=256 in the original paper and here), and an exhaustive search is performed to determine whether a feature lies within a given range [$\theta_{1},\;\theta_{2}$]. The weighted mean square error is applied as the optimal splitting criterion.

### 2.3. Filtering Steps

**Figure 3:**
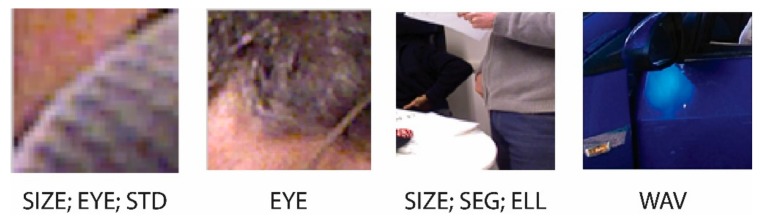
Examples of images rejected by the different filtering methods.

As noted in Figure 1, some of the false positives generated by the ensemble of classifiers are extracted by applying several filtering approaches that take advantage of the depth maps. The filters tested in this work are the set of six tested in [9] (viz. SIZE, STD, SEG, ELL, EYE, and SEC) and a new filter proposed here (viz. WAV), which is based on processing the image with different wavelets. Each of these filtering techniques is described below. Figure 3 illustrates images rejected by the seven types of filters.

#### 2.3.1. Image Size Filter (SIZE)

SIZE [10] rejects candidate faces based on the size of the face region extracted from the depth map. First, the 2D position and dimension ($W_{2D},\;h_{2D}$) in pixels of a candidate face region are identified by the face detector. Second, this information is used to estimate the corresponding 3D physical dimension in mm ($W_{3D},\;h_{3D}$) as follows:

(6) $$W_{3D}=W_{2D}\frac{\bar{d}}{f_{x}}\;\mathrm{and}\;h_{3D}=h_{2D}\frac{\bar{d}}{f_{x}},$$

where $f_{x}$ and $f_{y}$ are the Kinect camera focal lengths computed by the calibration algorithm in [57], and $\bar{d}$ is the average depth of the samples in the candidate bounding box. Face candidate regions are rejected when they lie outside the fixed range in cm [0.075, 0.35]. Note that $\bar{d}$ is defined as the median of the depth samples and is necessary for reducing the impact of noisy samples in the average computation.

#### 2.3.2. Flatness/Unevenness Filter (STD)

STD, as proposed in [9], extracts information from the depth map that relates to the flatness and unevenness of candidate face regions. Flat and uneven faces detected by the classifiers are then removed using the depth map and a segmentation method based on the depth map.

The filtering method is a two-step process. In Step 1, a segmentation procedure using the depth map is applied; in Step 2, the standard deviation (STD) of the pixels of the depth map that belong to the larger segment (i.e., the region obtained by the segmentaion procedure) is calculated from each face candidate region. Those regions whose STD lies outside the range of [0.01, 2.00] are rejected.

#### 2.3.3. Segmentation-Based Filtering (SEG and ELL)

SEG and ELL, proposed in [9], apply the segmented version of the depth image to compare its dimension to its bounding box in SEG or to its shape (which should approximate that of an ellipse) in ELL. From this information, two simple but useful evaluations can be made. In the case of SEG, the relative dimension of the larger area can be compared to the entire candidate image. The candidate regions where the area of the larger region is less than 40% of the entire area are rejected. In the case of ELL, the larger region is given a fitness score using the least-squares criterion to determine its closeness to an elliptical model. This score is calculated here using the MATLAB function fit_ellipse [61]. The candidate regions with a score higher than 100 are rejected.

#### 2.3.4. Eye-Based Filtering (EYE)

EYE, as proposed in [9], uses the presence of eyes in a region to detect a face. In EYE, two robust eye detectors are applied to candidate face regions [62,63]. Regions with a low probability of containing two eyes are rejected. 

One of the eye detectors [62] used in EYE is a variant of the Pictorial Structures (PS) model. PS is a computationally efficient framework that represents a face as an undirected graph $G=\left(V,\;E\right)$, where the vertices V correspond to facial features. The edges *E* describe the local pairwise spatial relationships between the feature set. PS is expanded in [62] so that it can deal with complications in appearance as well as with many of the structural changes that eyes undergo in different settings.

The second eye detector, presented in [63], makes use of color information to build an eye map that highlights the iris. A radial symmetry transform is applied to both the eye map and the original image once the area of the iris is identified. The cumulative results of this enhancement process provide the positions of the eye. Face candidates are rejected in those cases where detection of the eyes fall outside a threshold of 1 for the first approach [62] and of 750 for the second approach [63]. 

#### 2.3.5. Filtering Based on the Analysis of the Depth Values (SEC)

SEC, as proposed in [9], takes advantage of the fact that most faces, except those where people are lying flat, are on top of the body, while the remaining surrounding volume is often empty. With SEC, candidate faces are rejected when the neighborhood manifests a different pattern from that which is expected.

The difference in the expected pattern is calculated as follows. First, the rectangular region defining a candidate face is enlarged so that the neighborhood of the face in the depth map can be analyzed.

**Figure 4:**
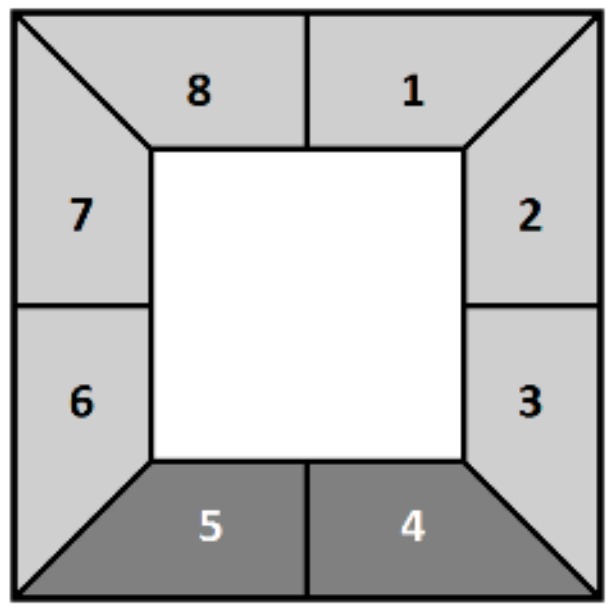
Examples of partitioning of a neighborhood of the candidate face region into 8 sectors (gray area). The lower sectors $Sec_{4}$ and $Sec_{5}$ that should contain the body are depicted in dark gray [9].

Second, the enlarged region is then partitioned into radial sectors (eight in this work, see Figure 4), each emanating from the center of the candidate face. For each sector $Sec_{i}$, the number of pixels $n_{i}$ are counted whose depth value $d_{p}$ is close to the average depth value of the face $\bar{d}$, thus:

(7) $$n_{i}=\left|\left\{p:|d_{p}-\bar{d|<t_{d}\wedge p\in Sec_{1}}\right\}\right|$$

where $t_{d}$ is a measure of closeness ($t_{d}=50$ cm here).

Finally, the number of pixels per sector is averaged on the two lower sectors ($Sec_{4}$ and $Sec_{5}$) and then again on the remaining sectors, from which two of the values, $n_{u}$ and $n_{l}$ respectively, are obtained. The ratio between $n_{u}$ and $n_{l}$ is then computed as:

(8) $$\frac{n_{l}}{n_{u}}=\frac{\frac{1}{2}\left(n_{4}+n_{5}\right)}{\frac{1}{6}\left(n_{1}+n_{2}+n_{3}+n_{6}+n_{7}+n_{8}\right)}.$$

If the ratio drops below a certain threshold, $t_{r}$ (where $t_{r}=0.8$ here), then the candidate face is removed. 

#### 2.3.6. WAV

WAV is a filtering technique that processes an image with different wavelets. With WAV, statistical indicators are extracted (e.g., the mean and variance) and used for discarding candidate images with no faces. Rejection is based on five criteria.

The first criterion applies phase congruency [64] to the depth map of the largest cluster, and the average value is used to discriminate between face/non-face. The segmentation process divides the image into multiple clusters, and only the largest cluster (that is, the one that is most likely to contain the face) is considered. Phase congruency has higher values when there are edges. WAV keeps only those candidates with an acceptable value, i.e., those with a number of edges that is neither too high nor too low, and deletes all others since they most likely contain no faces.

WAV is used here in two ways, but in both cases, Haar-like waves are selected since they often give the best results, as demonstrated in [65]. The first method (second criterion) works on the same principle as the phase congruency test: the Haar wave is applied to each image, and the average value is calculated for each one. However, the second test (third criterion) follows the approach in [50], where edge maps are first extracted and then fitted to an ellipse (the typical shape of a face). If an ellipse is found, then the image is rotated by an angle given by the intersection between the origin and the major axis of the ellipse, and the filter is applied to the rotated image. If no elliptical shape is found, the filter is applied to the original unrotated image. To conclude, the WAV filter produces higher values when it encounters specific features, especially abrupt changes that are typically not present in many non-faces.

Two remaining tests (fourth and fifth criteria) are based on Gabor’s logarithmic wavelet filter for finding the symmetry of the shape of the largest cluster. We calculate the phase symmetry of points in an image. This is a contrast invariant measure of symmetry [64]. High values indicate the presence of symmetry, which can mean the presence of a symmetrical shape, such as an ellipse, and therefore that have a good probability of containing a face. The first test discriminates based on the average of the scores, while the latter uses variance instead of the mean.

## 3. Results and Discussion

### 3.1. Datasets

Four datasets—Microsoft Hand Gesture (MHG) [66], Padua Hand Gesture (PHG) [67], Padua FaceDec (PFD) [10], and Padua FaceDec2 (PFD2) [9]—were used to experimentally develop the system proposed in this work. The faces in these datasets were captured in unconstrained environments. All four datasets contain colored images and their corresponding depth maps. All faces are upright and frontal with each possessing limited degrees of rotation. Originally, for two datasets, the faces were collected for gesture recognition rather than face detection. In addition, a separate set of images was collected for preliminary experiments and for parameter tunings. These faces were extracted from the Padua FaceDec dataset [10]. As in [9], these datasets were merged to form a challenging dataset for face detection.

In addition to the merged datasets, experiments are reported on the BioID dataset [56] so that comparisons with the system proposed here can be made with other face detection systems. Each of these five datasets is discussed below, with important information about each one summarized in Table 1.

MHG [66] was collected for the purpose of gesture recognition. This dataset contains images of 10 different people performing a set of gestures, which means that not only does each image in the dataset include a single face, but the images also exhibit a high degree of similarity. As in [9], a subset of 42 MHG images was selected, with each image manually labeled with the face position.

PHG [67] is a dataset for gesture recognition. It contains images of 10 different people displaying a set of hand gestures, and each image contains only one face. A subset of 59 PHG images were manually labeled.

PFD [10] was acquired specifically for face detection. PFD contains 132 labeled images that were collected outdoors and indoors with the Kinect 1 sensor. The images in this dataset contain zero, one, or more faces. Images containing people show them performing many different daily activities in the wild. Images were captured at different times of the day in vary lighting conditions. Some faces also exhibit various degrees of occlusion.

PFD2 [9] contains 316 images captured indoors and outdoors in different settings with the Kinect 2 sensor. For each scene, a 512 × 424 depth map and a 1920 × 1080 color image were obtained. Images contain zero, one, or more faces. Images of people show them in various positions with their heads tilted or next to objects. The outdoor depth data collected by Kinect 2 are highly noisy compared to the images collected with Kintect 1. This makes PFD2 an even more challenging dataset. The depth data was retroprojected over the color frame and interpolated to the same resolution to obtain two aligned depth and color fields.

The MHG, PHG, PFD, and PFD2 datasets were merged, as in [9], to form a larger, more challenging dataset, called MERGED, containing 549 images with 614 total faces. Only upright frontal faces with a maximum rotation of ±30° were included. Parameter optimization of the face detectors was manually performed and fixed for all images even though they came from four datasets with different characteristics.

As a final dataset for validating the approach proposed in this work, we chose one of the leading benchmark datasets for upright frontal face detection: the BioID dataset [56]. It contains 1521 images of 23 people collected during several identification sessions. The images in BioID are gray-scale and do not include depth map information. Moreover, the degree of rotation in the facial images is small. As a consequence, most of the filters applied to the ensembles were not transferable to the BioID dataset. Despite this shortcoming, this dataset is useful in demonstrating the effectiveness of the ensembles developed in this work.

### 3.2. Performance Indicators

The following two well-known performance indicators are reported here:

- Detection rate (DR): the ratio between the number of faces correctly detected and the total number of faces in the dataset. The faces were manually labeled. DR is evaluated at different precision levels considering different values of “eye distance”. Let $d_{l},\left(d_{r}\right)$ be the Euclidean distance between the manually extracted $C_{l},\left(C_{r}\right)$ and the detected $C\prime_{l},\left(C\prime_{r}\right)$ left (right) eye positions. The relative error of detection is defined as $ED=\max\left(d_{l},d_{r}\right)/d_{lr}$, where the normalization factor $d_{lr}$ is the Euclidean distance of the expected eye centers used to make the measurement independent of the scale of the face in the image and of the image size. There is a general agreement [56] that ED ≤ 0.25 is a good criterion for claiming eye detection, since this value roughly corresponds to an eye distance smaller than the eye width. Some face detectors (i.e., FL and RF) give the positions of the eye centers as the output, whereas for others (i.e., VJ and SN), the eye position is assumed to be a fixed position inside the face bounding box.
- False positives (FP): the number of candidate faces that do not include a face.

### 3.3. Experiments

The first experiment compares the detection rates of the six face detectors, along with some of their combinations, by adjusting (1) the sensitivity values of s, where applicable, and (2) the detection procedure which either does or does not involved the addition of poses constructed by rotating images 20°/−20°.

The value for the sensitivity threshold s is shown in parentheses in Table 1. To reduce the number of false positives (FP), all output images having a distance of their centroid ≤30 pixels are merged as in [9].

As evident in the results in Table 2, the addition of rotated poses is of little value for the RF face detector, since this detector was originally trained on images that contained rotated faces. Thus, the addition of rotated poses increased the number of false positives.

Only the most interesting results are reported for the ensembles of classifiers. As can be seen in Table 2, high-performing approaches in an ensemble increase the detection rates while also generating more false negatives.

In Table 3, the performance of the face detectors presented in Table 2 are reported on the BioID dataset. As noted in [9], the addition of rotated poses is not needed when images are acquired in constrained environments. Although there is no significant difference in performance when adding the rotated poses, a difference is evident in the number of false positives that the rotated poses produce: they increase the false positives. 

In Table 3, we also discover that each of the face detectors identifies a different set of faces. This diversity in the individual face detectors is what enables the ensemble to improve the best standalone approaches. It is also noteworthy that the same classifier can perform differently on the MERGED versus BioID dataset. For instance, RF works well on BioID but not so well on MERGED; perhaps this is because it contains low-quality faces.

In Table 4, an experiment is reported that evaluated the seven filtering steps, as detailed in Section 2.3, along with their combinations. The first experiments showed that the best ensemble (considering the trade-off between performance and false positives) is FL + RF(−0.65) + SN(1)* + SFD. For this reason, the filtering sets are tested only for this detector.

SIZE is clearly the best method for removing false positive candidates from a set of faces detected by FL + RF(−0.65) + SN(1)* + SFD. The next best filter is EYE. However, because EYE is computationally expensive, it cannot be used in all applications. Although the other filters, when considered individually, are of less value because of their low computational costs, they are useful for reducing the number of false positives when applied sequentially. If real-time detection is not required (which is typically the case when tagging faces), then EYE filtering can be used to reduce the number of false positives produced by an ensemble without decreasing the number of true positives.

The results presented in the previous tables shows that the proposed approach performs better than FL and SPD, both of which are considered two of the best face detectors in the literature. It is true that the results reported here have been obtained on two rather small datasets; nonetheless, MERGED is highly realistic. Thus, it is reasonable to predict that the best ensemble proposed in this work would perform comparatively well in real-world conditions. The images contained in MERGE include those containing a single frontal face as well as those containing multiple faces acquired “in the wild”.

Finally, in order to evaluate the computational cost of our approach, the processing time per 640 × 480 image on a i7-7700HQ PC system is reported in Table 5 for each detection method of “FL* + RF(−0.65) + SN(1)* + SFD” and each additional filter (on a candidate region of size 78 × 78 pixels). All the tests are performed without parallelizing the code. However, it should be noted that the filters and face detectors can run in parallel, resulting in a significant reduction of computation time.

## 4. Conclusions

In this paper, an ensemble of state-of-the-art face detectors is combined with a set of filters calculated from both the depth map and the color image. The filters reduce the number of false positives produced by the ensemble while maximizing the detection rate. A set of seven filters based on the size, the flatness, or the unevenness of the candidate face regions, or on the size of the larger cluster of the depth map of the candidate face regions, or on eye detection or the degree of ellipse fitting are evaluated, including a new method proposed here that is based on processing the candidate region with different wavelets. The method proposed in this work for developing an ensemble of face detectors uses the depth map to obtain increased effectiveness even under many indoor and outdoor illumination settings.

The experimental results demonstrate that the filtering steps significantly reduce the number of false positives (from 16,325 to 1018) without significantly decreasing the detection rate (from 92.02 to 90.07) on a challenging dataset containing images with cluttered and complicated backgrounds. The performance of the proposed system is also reported on the challenging BioID benchmark to validate the approach presented here further and to compare the best performing ensemble with the state-of-the-art in face detection.

The face detector named SFD is shown to outperform all other standalone methods. However, an ensemble proposed here that combines SFD with other types of face detectors is shown to boost the standalone performance of SFD. Obviously, increasing the number of face detectors included in ensembles increases the number of false positives; however, as the experiments in this work demonstrate, the application of a new cascade of filters reduces this number to acceptable levels.

## Figures and Tables

**Table 1:** Characteristics of the six datasets. MHG: Microsoft Hand Gesture, PHG: Padua Hand Gesture, PFD: Padua FaceDec, and PFD2: Padua FaceDec2.

Dataset	Number Images	Color Resolution	Depth Resolution	Number Faces	Difficulty Level
MHG	42	640 × 480	640 × 480	42	Low
PHG	59	1280 × 1024	640 × 480	59	Low
PFD	132	1280 × 1024	640 × 480	150	High
PFD2	316	1920 × 1080	512 × 424	363	High
MERGED	549	---	---	614	High
BioID	1521	384 × 286	---	1521	High

**Table 2:** Performance of the six face detectors and the best performing ensembles (see the last seven rows) on the MERGED dataset (* denotes the addition of the 20°/−20° rotated images/poses in the dataset). As in [9], a face is considered detected in an image if the eye distance ED<0.35. DR: detection rate, FL: fast localization, FP: false positives, NPD: normalized pixel difference, SFD: Single Scale-invariant Face Detector, SN: Split up sparse Network of Winnows, VJ: Viola–Jones.

Face Detector(s)/Ensemble	+Poses	DR	FP
VJ(2)	No	55.37	2528
RF(−1)	No	47.39	4682
RF(−0.8)	No	47.07	3249
RF(−0.65)	No	46.42	1146
SN(1)	No	66.61	508
SN(10)	No	46.74	31
FL	No	78.18	344
NPD	No	55.70	1439
SFD	No	81.27	186
VJ(2) *	Yes	65.31	6287
RF(−1) *	Yes	49.67	19,475
RF(−0.8) *	Yes	49.67	14,121
RF(−0.65) *	Yes	49.02	5895
SN(1) *	Yes	74.59	1635
SN(10) *	Yes	50.16	48
FL *	Yes	83.39	891
NPD *	Yes	64.17	10,431
FL + RF(−0.65)	No	83.06	1490
FL + RF(−0.65) + SN(1)	No	86.16	1998
FL + RF(−0.65) + SN(1) *	Mixed	88.44	3125
FL * + SN(1) *	Yes	87.79	2526
FL * + RF(−0.65) + SN(1) *	Mixed	90.39	3672
FL * + RF(−0.65) + SN(1) * + SFD	Mixed	91.21	3858
FL * + RF(−0.65) + SN(1) * + NPD * + SFD	Mixed	**92.02**	16,325

**Table 3:** Performance of the six face detectors and ensembles reported above on the BioID dataset (note: some values are taken from [9]).

Face Detector(s)/Ensemble	+Poses	DR (ED < 0.15)	DR (ED < 0.25)	DR (ED < 0.35)	(FP)
VJ(2)	No	13.08	86.46	99.15	517
RF(−1)	No	87.84	98.82	99.08	80
RF(−0.8)	No	87.84	98.82	99.08	32
RF(−0.65)	No	87.84	98.82	99.08	21
SN(1)	No	71.27	96.38	97.76	12
SN(10)	No	72.06	98.16	99.74	172
FL	No	92.57	94.61	94.67	67
SFD	No	99.21	99.34	99.34	1
VJ(2) *	Yes	13.08	86.46	99.15	1745
RF(−1) *	Yes	90.53	99.15	99.41	1316
RF(−0.8) *	Yes	90.53	99.15	99.41	589
RF(−0.65) *	Yes	90.53	99.15	99.41	331
SN(1) *	Yes	71.33	96.52	97.90	193
SN(10) *	Yes	72.12	98.36	99.87	1361
FL *	Yes	92.57	94.61	94.67	1210
FL + RF(−0.65)	No	98.42	99.74	99.74	88
FL + RF(−0.65) + SN(10)	No	99.15	99.93	99.93	100
FL + RF(−0.65) + SN(1) *	Mixed	99.15	100	100	281
FL * + SN(1) *	Yes	98.03	99.87	99.93	260
FL * + RF(−0.65) + SN(1) *	Mixed	99.15	100	100	1424
FL * + RF(−0.65) + SN(1) * + SFD	Mixed	**99.41**	**100**	100	1425

**Table 4:** Performance of FL + RF(−0.65) + SN(1)* + SFD obtained combining different filtering steps on MERGED.

Filter Combination	DR	FP
SIZE	91.21	1547
SIZE + STD	91.21	1514
SIZE + STD + SEG	91.21	1485
SIZE + STD + SEG + ELL	91.04	1440
SIZE + STD + SEG + ELL + EYE	90.55	1163
SIZE + STD + SEG + ELL + SEC + EYE	90.39	1132
SIZE + STD + SEG + ELL + SEC + EYE + WAV	90.07	1018

**Table 5:** Average processing time per image in ms.

Detection Method/Filter	ms
RF	12,571
SN	1371
FL	170
SPD	175
SIZE	0.33
STD	10.86
SEG	8.808
ELL	10.24
EYE	19,143
WAV	179.4
